# Treatment of microcirculation dysfunction in type 2 diabetic mellitus with Shenqi compound prescription: A protocol of systematic review and meta-analysis of randomized clinical trials

**DOI:** 10.1097/MD.0000000000022347

**Published:** 2020-10-09

**Authors:** Min Zhong, Xiaohan Song, Xinxia Zhang, Junmin Chen, Lizhen Wang, Jia Xia, Xiaoming Tang, QI Chen, Botong Yang

**Affiliations:** a Hospital of Chengdu University of Traditional Chinese Medicine; b Chengdu University of Traditional Chinese Medicine, Chengdu, Sichuan, China.

**Keywords:** meta analysis and systematic review, microcirculation dysfunction in type 2 diabetic mellitus, protocol, shenqi compound prescription

## Abstract

**Introduction:**

Type 2 diabetic mellitus (T2DM) is a chronic disease. In 2013, the International Diabetes Federation showed that the total number of diabetic patients aged 20 to 79 years in China was 89 million, and it is expected to increase to 143 million by 2035.^[1]^ The incidence of T2DM and its complications in patients with blood glucose is gradually increasing, and there are low awareness rate, low diagnosis rate and high disability rate, which has become a global public health problem. Microcirculation Dysfunction in Type 2 diabetic mellitus (MDT2DM) plays an important role in the development of diabetic nephropathy, diabetic retinopathy, diabetic neuropathy and diabetic foot disease. It is 1 of the common etiological mechanisms of diabetic chronic complications. Patients with MDT2DM, serious complications, increase the quality of life of patients with social impact. Diabetic lower extremity microcirculation disease (dlemd) is the main cause of the occurrence, development and difficult healing of diabetic foot. Microvascular disease is microcirculation dysfunction. It has been proved that Shenqi compound prescription can treat T2DM macrovascular disease and microvascular dysfunction. However, due to the lack of evidence and no specific methods or suggestions, it is necessary to conduct a systematic evaluation of Shenqi compound prescription to provide effective evidence for further research.

**Methods and analysis:**

The following databases will be searched from their inception to August 2020: Electronic database includes PubMed, Embase, Cochrane Library, Web of Science, Nature, Science online, Chinese Biomedical Database WanFang, VIP medicine information, and China National Knowledge Infrastructure.

**Primary outcomes:**

superoxide dismutase, malondialdehyde, C-reactiveprotein, HOMA-IR, advanced glycation end products, FPG, 2hBG, glycosylated hemoglobinA1c, fasting insulin ;

**Additional outcomes:**

low density lipoprotein, high density lipoprotein, triglycerides, total serum cholesterol. Data will be extracted by 2 researchers independently, risk of bias of the meta-analysis will be evaluated based on the Cochrane Handbook for Systematic Reviews of Interventions. All data analysis will be conducted by data statistics software Review Manager V.5.3. and Stata V.12.0.

**Results:**

The results of this study will systematically evaluate the efficacy and safety of Shenqi compound prescription in treating patients with MDT2DM

**Conclusion:**

The systematic review of this study will summarize the current published evidence of Shenqi compound prescription in the treatment of MDT2DM, and further guide its popularization and application.

**Ethics and dissemination:**

This study is a systematic review, the outcomes are based on the published evidence, so examination and agreement by the ethics committee are not required in this study. We intend to publish the study results in a journal or conference presentations.

**Open Science Fra mework (OSF) registration number:**

August 24, 2020.osf.io/es6z7. (https://osf.io/es6z7)

## 1 Introduction

According to the epidemiological survey results of type 2 diabetic mellitus (T2DM) in China, as of 2013, the prevalence of chronic diseases and its risk factors in China showed that the prevalence of diabetes in people aged 18 and above was 10.4%.^[2]^ Diabetic complications can be divided into acute complications and chronic complications. Chronic diabetic complications mainly refer to macrovascular complications, microvascular complications and neuropathy in diabetic patients;^[3]^ Among them, diabetic microvascular complications include diabetic nephropathy (DN), diabetic retinopathy (DR),diabetic neuropathy,diabetic foot disease (DR), and so on. The epidemiological survey of type 2 diabetes in large cities in China shows that the prevalence of microvascular complications is 39.7% of DN and diabetes. Retinopathy is 31.5%, the combined central and peripheral neuropathy is about 61.8%, and the prevalence of diabetic foot is 15%.^[4]^ The prevalence of diabetes has brought a serious social and economic burden, and the global diabetes related health expenditure reached 727 billion US dollars in 2017. Microcirculation is the blood circulation between arterioles and venules. It is the basic structure and functional unit of material exchange between blood and tissues.microvascular disease namely microvascular Dysfunction, refers to vascular cell dysfunction in inflammatory reaction, metabolic disorders and other pathological conditions, leading to increased vascular permeability, abnormal autonomic nerve function, endocrine hormone secretion disorder, resulting in its inability to adapt to the metabolic level of tissues, affecting the material exchange of tissues and organ function. It plays an important role in the occurrence and development of DN, DR, diabetic peripheral neuropathy, and diabetic foot disease. It is 1 of the important physiological and pathological bases of chronic diabetic complications, and is also 1 of the common etiological mechanisms of diabetic chronic complications.^[5]^ Microcirculation dysfunction in type 2 diabetic mellitus (MDT2DM) is caused by diabetes related factors and is a common chronic complication of diabetes. One of the same etiological mechanisms is also 1 of the important physiological and pathological basis of chronic diabetic complications. It not only plays a role in diabetic vascular complications, but also participates in the occurrence and development of insulin resistance and diabetes mellitus. Due to microcirculation dysfunction (MD), the ability to supply oxygen to tissues through capillaries is decreased, and long-term hypoxia accelerates vascular endothelial injury,^[6]^ abnormal Hemorheology and excessive oxidative stress of cells, resulting in increased production of reactive oxygen species, reduced production of nitric oxide (no) in vascular endothelial cells, and decreased sensitivity of vascular smooth muscle intima to no^[7,8]^ Further affect the microcirculation function, so as to accelerate the progress of the disease. MD not only play a role in diabetic vascular complications, but also participate in the occurrence and development of insulin resistance and diabetes, throughout diabetes mellitus.^[9,10]^ Studies have confirmed that T2DM microangiopathy is the joint action of hyperglycemia, insulin resistance, lipid metabolism disorder, various cytokines and other inflammatory factors.^[11–13]^ Moreover, the onset of T2DM microangiopathy is insidious, and there is no obvious clinical symptoms in the early stage. At present, there are different detection indexes for evaluating MDT2DM at home and abroad. involving many risk factors and detection indexes, but still lack of specificity; and the treatment methods of microvascular complications at home and abroad are different, mainly including dilation of blood vessels, improvement of hemodynamics, protection of vascular endothelial related drugs and some traditional Chinese medicine preparations However, the selection and use of related drugs are still not standardized. Many drugs to improve MD still lack of evidence-based medicine evidence. Especially, the safety and effectiveness of Chinese medicine preparations in the treatment of MD are still lack of research data support, lack of standardized and effective prevention and treatment methods, which brings serious economic burden to individuals, families and society In recent years, traditional Chinese medicine has been widely used in clinical and experimental research of MDT2DM, and its effectiveness has been fully proved. A large number of clinical trials and animal experiments show that Shenqi compound prescription has a good effect on controlling blood glucose and improving the symptoms of MDT2DM, but its effectiveness and safety have not been clearly concluded. Therefore, this study intends to use the systematic evaluation and meta-analysis methods of Shenqi compound prescription in the treatment of MDT2DM to evaluate its efficacy and safety.

## 2 Methods

### 2.1 Study registration

The protocol has been registered in OSF (Open Science Framwork) Preregistration. August 24, 2020.osf.io/es6z7. (https://osf.io/es6z7) The protocol will follow the statement guidelines of Preferred Reporting Items for Systematic Reviews and Meta-Analyses Protocols (PRISMAP), Changes will be reported in the full review as required.

### 2.2 Inclusion and exclusion criteria for study selection

#### 2.2.1 Inclusion criteria

Inclusion criteria are all randomized controlled trials (RCTs). Shenqi compound prescription Chinese herbal medicine was mainly used in the treatment of MDT2DM. The language of the trials to be included only Chinese or English.

#### 2.2.2 Exclusion criteria. Following stuides will be excluded

(1) Type I diabetes, ultrasound examination has obvious lower extremity artery stenosis, serious liver and kidney, blood system diseases.
(2) the treatment was combined with other treatment other than chinese herbs.
(3) Non-RCTs and Quasi-RCTs
(4) Case series and Reviews
(5) Animal studies

Note: all the above items are excluded.

### 2.3 Types of participants

Types of subjects included patients diagnosed with MDT2DM, regardless of the degree. All patients should be treated with Shenqi compound prescription, or combined with other conventional treatment methods. There is no concept of gender, race or education.

### 2.4 Experimental interventions

Traditional Chinese medicine Shenqi compound prescription intervention is the main treatment. Western medicine and traditional Chinese medicine preparations with definite improvement of microcirculation should not be used, such as high-dose aspirin (more than 300 mg/d), antiplatelet aggregation, beta, thiazolidinediones, statins, Ginkgo biloba extract, Fufangdanshen dripping pills, Panax notoRenshen preparations.

### 2.5 Control interventions

Interventions may include: No treatment, The placebo, Non-drug interventions (eg diet, exercise, etc), Conventional western medicine hypoglycemic drugs (eg, metformin, euglycemic, etc), Insulin (any kind of insulin). Combined interventions are allowed as long as all groups in the randomized trial receive the same combined intervention.

### 2.6 Types of outcome measures

#### 2.6.1 Main outcomes

1. superoxide dismutase;
2. malondialdehyde;
3. C-reactiveprotein ;
4. HOMA-IR;
5. advanced glycation end products;
6. Fasting insulin;
7. glycosylated hemoglobinA1c;
8. fasting blood-glucose;
9. 2hours-post prandial blood glucose.

#### 2.6.2 Additional outciomes

(1) low density lipoprotein;
(2) high density lipoprotein;
(3) triglycerides;
(4) total serum cholesterol.

## 3 Data sources

### 3.1 Electronic searches

The following data bases will be searched to identify eligible studies: PubMed, Embase, Cochrane Library, Web of Science, Nature, Science on line, Chinese Biomedical Database WanFang, VIP medicine information, and China National Knowledge Infrastructure. The time range is: the starting time is determined according to the first literature available, and the deadline is August 2020.

### 3.2 Other search resources

**Figure 1:**
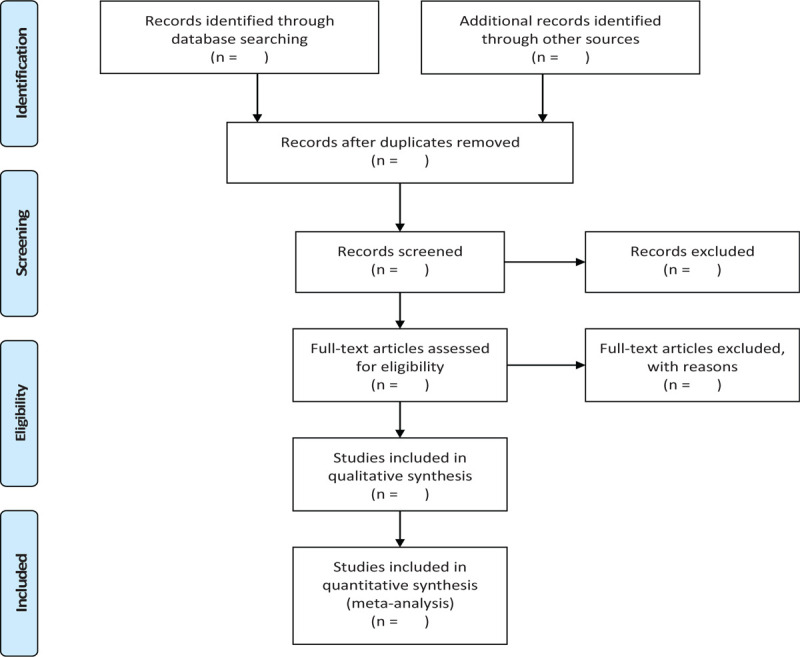
The research flowchart. This figure shows the identification, screening, eligibility and included when we searching articles.

In order to get more complete evidence, we will also retrieve other related documents by manually, such as medical textbooks, clinical laboratory manuals and so on. If it is necessary, we will contact with trail author to obtain the latest clinical data. Moreover, studies associated with the review will be identified via evaluating related conference proceedings. The research flowchart is shown in Figure 1.

### 3.3 Search strategy

The following search terms will be used:

randomized controlled trial/RCT; Type 2 diabetic mellitus/T2DM; MD in Type 2 diabetic mellitus/Microcirculation disturbance in Diabetes /Microvascular disease, Type2, traditional chinese medicine/TCM;Shenqi compound prescription/Shenqi compound,different retrieval strategies in Chinese and foreign databases will be used. Language restrictions are Chinese and English. There is no publication restriction. Here we take the search strategy in PubMed as an example and list in Table 1.

**Table 1:**
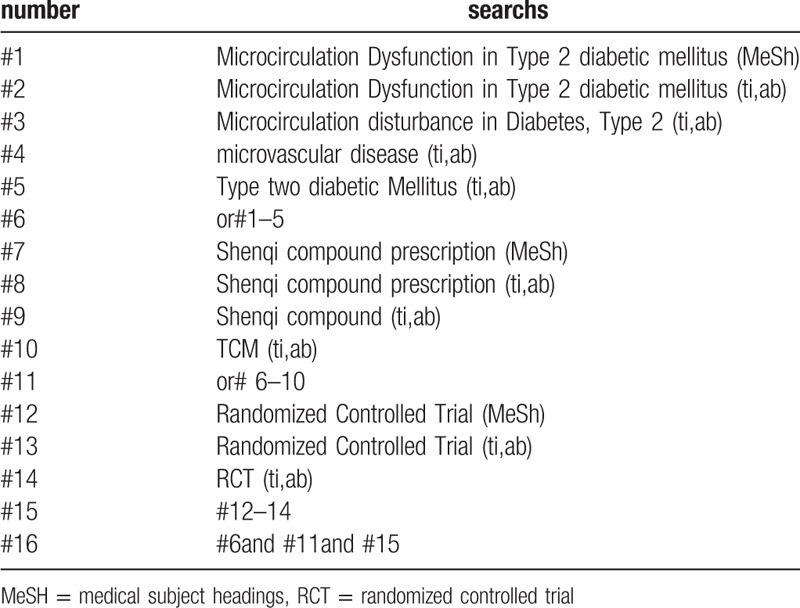
The research strategy. Table 1 Search stragtegy sample of PubMed.

## 4 Data collection and analysis

### 4.1 Study selection

All articles in the search results were independently evaluated by 2 independent researchers (ZM, SXH) according to inclusion and exclusion criteria. Reviewers will then independently extract and collect the data included in the study using pre-designed data collection forms. Discrepancies will be discussed and resolved by consensus with a third author (YR).

### 4.2 Data extraction and management

The following informations will be extracted from each study:

(1) Normal test characteristics: title, author, year.
(2) baseline data: sample size, age, gender, diagnosticcriteria, course of disease.
(3) interventions: Shenqi compound, control of intervention details, intervention.

If the information is not enough, we will contact experts and authors in this field to get relevant information.

### 4.3 Assessment of the reporting quality and risk of bias

The risk of bias will be assessed by 2 independent authors (M Z and XH S), together with completing the STRICTA checklist. The Cochrane System Evaluator's Manual give the evaluation criteria for authors to evaluated the RCTs’ quality. Assessing the risk of bias:

(1) random sequence generation;
(2) allocation concealment;
(3) blinding of participants and personnel;
(4) blinding of outcome assessment;
(5) incomplete outcome data;
(6) selective outcome reporting;
(7) other bias.

Any disagreement will be discussed or consulted with a third reviewer. Each them will be described from 3 levels: “high risk”, “low risk”and “unclear”.

### 4.4 Measures of a treatment effect

The dichotomous outcomes will be expressed by the Odds ratio (ORs), while the continuous data will use the Standardized mean difference (SMD). All these outcomes report 95% confidence interval.

### 4.5 Management of missing data

We will take the method of contacting corresponding authors to obtain the missing data. If there is no response, we will analyze only the available data and describe the reason and impact of this exclusion in the paper.

### 4.6 Assessment of a reporting bias

The bias of publication will be explored through funnel plot analysis. If the funnel plot show asymmetry, it will be evaluated via the Egger and Beg tests, and *P* value < .05 means the publication bias is significant.

### 4.7 Assessment of heterogeneity

There are 2 main methods for testing heterogeneity, namely graphical method (funnel plot, forest plot) and statistical test (*Q* value statistic test, *I*^2^ statistic test, *H* statistic test). The *I*^2^ statistic test method shows us When *I*^2^ is 0, it means that studies are completely homogeneous, If *I*^2^ > 50%, it indicates there is heterogeneity in studies.

### 4.8 Data synthesis and grading of quality of evidence

The results of the study will be analyzed by RevMan 5.0 software provided by Cochrane collaborate on network. The binary data will be expressed by the odds ratio, while the continuous data will use the mean difference. To test the heterogeneity of the research results, when the *I*^2^ < 50% or *P* > .1, the heterogeneity is significant. The random effect model was used for the meta-analysis, otherwise, we choose the fixed effect model.

### 4.9 Subgroup analysis

XXX

### 4.10 Sensitivity analysis

Sensitivity analysis can not only assess the stability and reliability of the conclusions of the Meta analysis, but also assess whether the changes in the results are related to the impact of a single study. If the stability of the conclusion is poor, we can achieve When the heterogeneity test results are heterogeneous, we need to clarify the source of the heterogeneity by subgroup analysis. The effects of different types of therapy including design scheme, severity of illness, age, sex, and mild or severe MDT2DM were analyzed. We will also delete low-quality and/or medium-quality studies to check the robustness of the results.

The purpose of increasing stability by changing the analysis model, inclusion and exclusion criteria, or excluding a certain type of literature.

### 4.11 Ethics and dissemination

We will publish the system review results in peer-reviewed journals, disseminated in meetings or in peer-reviewed publications. Aggregated published data will be used to exclud data of individuals, so there is no need for obtaining the ethical approval or patients’ informed consent.

## 5 Discussion

Shenqi compound prescription can be used for a variety of metabolic diseases and related complications, such as DN, a large number of clinical studies have shown that vascular endothelial damage is the initial factor causing vascular inflammatory reaction and atherosclerotic plaque formation. Shenqi compound preparation can inhibit oxidative stress, thereby reducing vascular endothelial damage, reducing the production of inflammatory factors, and alleviating MD. Previous studies have found that Shenqi compound prescription is an effective way to reduce vascular endothelial damage and inflammatory factors The compound preparation can reduce blood glucose and lipid, promote insulin secretion, improve glucose metabolism, improve IR, improve hemodynamics, reduce pathological angiogenesis, inhibit atherosclerosis and myocardial fibrosis, maintain smooth blood flow, and improve hemodynamics.^[14]^ From the source to correct the body's metabolic imbalance, while regulating blood glucose and lipid metabolism, significantly improve MDT2DM, improve the quality of life of patients.

The effective effect of Shenqi compound prescription on MDT2DM may be related to its main active ingredients and compatibility of traditional Chinese medicine

Shenqi compound prescription is composed of Rensheng 10 g, Huangqi 30 g, Shanyao15 g, Shanzhuyu 15 g, Shengdihuang 15 g, Tianhuafen 10 g, Danshen15 g, Zhidahuang 5 g, etc.

Modern pharmacology has proved that Renshen, Renshen polypeptides, Renshen non saponins and other water extracts have the effect of reducing blood sugar. The mechanism is to promote glycogen decomposition, inhibit lactic acid synthesis of glycogen and promote the aerobic oxidation of sugar^[15]^ Huangqi has the functions of invigorating the spleen and benefiting the middle, strengthening the body and diuresis. Animal experiments show that the extract of Huangqi polysaccharide has a bidirectional regulating effect on blood glucose in mice;^[16]^ the compatibility of Renshen and Huangqi can greatly tonify spleen qi and improve hemorheology. Shanyao has the effect of invigorating the spleen and stomach, promoting body fluid and benefiting lung. Clinical studies have confirmed that shanyao can regulate blood glucose by promoting insulin secretion and improving isletβcell function^[17]^ Shanzhuyu has the effect of Tonifying the liver and kidney, astringent and astringent; the components of Shengdi huang extract Catalpol, Rehmannia oligosaccharide, Rehmannia glycoside and other components have obvious hypoglycemic and regulatory effects; Tianhuafen has the effect of clearing away heat and fire, generating fluid and relieving thirst, and the pharmacological effect of Tianhuafen is mainly antioxidant, reducing vascular endothelial damage, and reducing blood glucose.^[18,19]^ Danshen contains tanshinone, polysaccharide compounds, etc., which can effectively inhibit the release of inflammatory factors, reduce oxygen free radicals, achieve anti-inflammatory and anti-oxidation effects, thus reducing microvascular lesions;^[20]^ Zhidahuang can remove oxygen free radicals,Zhidahuang can also improve effective blood volume, reduce blood viscosity, and help to relieve MD;^[21]^ Danshen and Zhidahuang both have the effect of improving blood flow. Early application of Shenqi compound prescription can correct the metabolic imbalance from the source of MDT2DM, improve the antioxidant capacity of human body, inhibit oxidative stress, reduce the generation of oxygen free radicals and metabolites, promote the excretion of metabolites and block the “metabolic memory” effect, so as to prevent and treat the microvascular complications of T2DM. Therefore, it is suggested that traditional Chinese medicine should be considered in clinical medication rather than chemical extracts to accurately reflect the functional value of traditional Chinese medicine and its compound.

In conclusion, systematic review and meta-analysis are helpful to determine the potential value of Shenqi compound prescription in the treatment of MDT2DM This study can not only provide the basis for the release of diabetes treatment guidelines, but also promote the application of traditional Chinese medicine prescriptions, so that more patients benefit.

## Author contributions

**Conceptualization**: Min Zhong, Xiaohan Song Xinxia Zhang.

**Data curation**: Junmin Chen, Lizhen Wang, Xiaoming Tang.

**Formal analysis**: Min Zhong, Jia Xia.

**Methodology**: Min Zhong, Qi Chen, Xinxia Zhang.

**Project administration**: Xinxia Zhang.

**Resources**: Min Zhong, Botong Yang.

**Software**: Min Zhong.

**Supervision**: Lizhen Wang.

**Writing – original draft**: Min Zhong.

**Writing – review & editing**: Xinxia Zhang.

## Abbreviations

Abbreviations: DN = diabetic nephropathy, DR = diabetic-retinopathy, MD = microcirculation dysfunction, MDT2DM = Microcirculation Dysfunction in Type 2diabetic mellitus, T2DM = type 2 diabetic mellitus.
